# Transabdominal preperitoneal repair for an adolescent patient with Hunter syndrome: a case report

**DOI:** 10.1186/s40792-019-0645-2

**Published:** 2019-05-31

**Authors:** Yoichiro Tada, Manabu Yamamoto, Teppei Sunaguchi, Chihiro Uejima, Akimitsu Tanio, Yuki Murakami, Shuichi Takano, Teruhisa Sakamoto, Soichiro Honjo, Keigo Ashida, Hiroaki Saito, Yoshiyuki Fujiwara

**Affiliations:** Division of Surgical Oncology, Department of Surgery, School of Medicine, Tottori University Faculty of Medicine, 36-1 Nishi-cho, Yonago, 683-8504 Japan

**Keywords:** Hunter syndrome, An inguinal hernia, Transabdominal preperitoneal repair

## Abstract

**Background:**

Hunter syndrome is an X-linked disorder caused by a deficit of the lysosomal enzyme iduronate-2-sulfatase and is associated with many disorders. Patients with Hunter syndrome often develop inguinal hernias in early childhood and undergo Potts’ method, laparoscopic percutaneous extraperitoneal closure (LPEC), or laparoscopic direct suture.

**Case presentation:**

An 18-year-old male visited our hospital for evaluation of a palpable mass in the right groin hernia. Computed tomography revealed a right indirect inguinal hernia. He had a history of repeated admission to our hospital and pediatric treatments for pneumonia, heart failure, and convulsions after birth. Because he has stopped growing and a wide hernia orifice was present with no apparent hernia on the left side, we performed TAPP repair. During surgery, we noted softness of the abdominal wall, similar to children’s abdominal wall, and laparoscopy revealed well-developed veins around the spermatic cord and testicular artery. The softness of the abdominal wall made insertion of the trocars difficult and well-developed veins needed our special care to avoid hemorrhage. After surgery, the patient developed a convulsion due to Hunter syndrome and subsequent aspiration pneumonia; however, he recovered with medical treatments administered in cooperation with specialists and was discharged on postoperative day 9.

**Conclusion:**

This is the first reported patient with Hunter syndrome whose inguinal hernia was treated by TAPP repair. TAPP repair might be a useful procedure even for adolescent patients with Hunter syndrome, although adequate care is needed for symptoms due to Hunter syndrome.

## Background

In 1917, Hunter reported a rare familial disease in two brothers [1]. This disease was called Hunter syndrome and is an X-linked genetic disorder characterized by lack of the lysosomal enzyme iduronate-2-sulfatase [2]. Approximately 200 patients with Hunter syndrome exist in Japan, and their condition is characterized by developmental disabilities such as joint stiffness, gargoyle-like facies, respiratory infections, heart failure, and inguinal hernias. Lin et al. [3] reported that most patients with Hunter syndrome underwent hernia repair in early childhood by Potts’ method [4], laparoscopic percutaneous extraperitoneal closure (LPEC) [5], or laparoscopic direct suture [6, 7]. We herein report a very rare case involving an 18-year-old patient with Hunter syndrome who underwent transabdominal preperitoneal (TAPP) repair for an indirect inguinal hernia.

## Case presentation

**Fig. 1:**
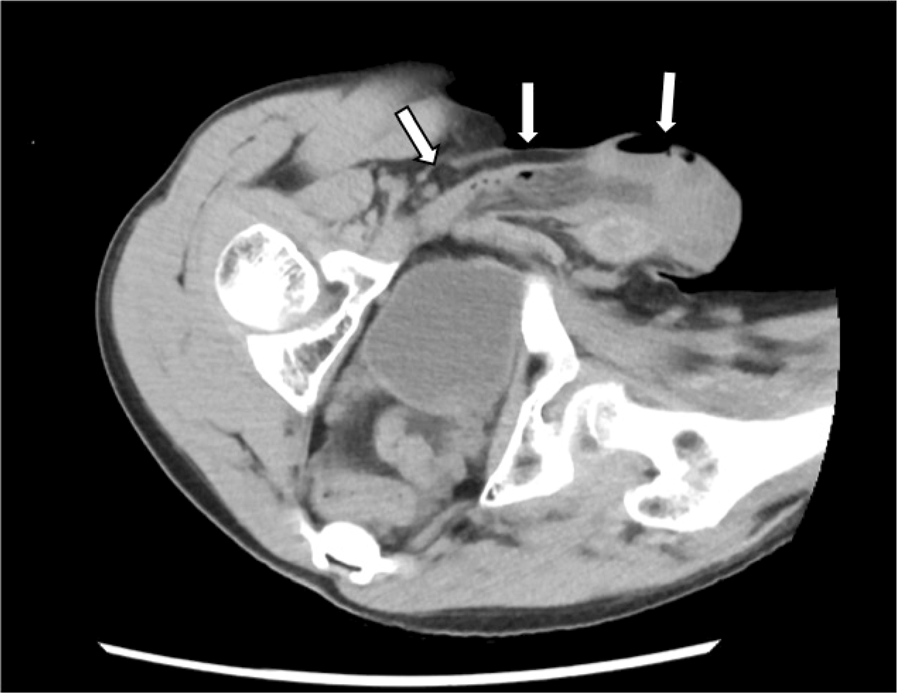
Computed tomography showed an indirect inguinal hernia on the right side

An 18-year-old male patient with Hunter syndrome visited at the Department of Surgery at Tottori University Hospital for evaluation of a palpable mass in the right groin area. Computed tomography revealed a right indirect inguinal hernia containing the small intestine (Fig. 1). He had a history of repeated admission to our hospital and pediatric treatments for pneumonia, heart failure, and convulsions after birth. He was small in stature (height, 133.7 cm; weight, 36.6 kg). We observed no apparent hernia on the left side; however, a wide hernia orifice was present. Therefore, we selected a laparoscopic approach instead of an anterior approach to check for an occult hernia on the left side.

**Fig. 2:**
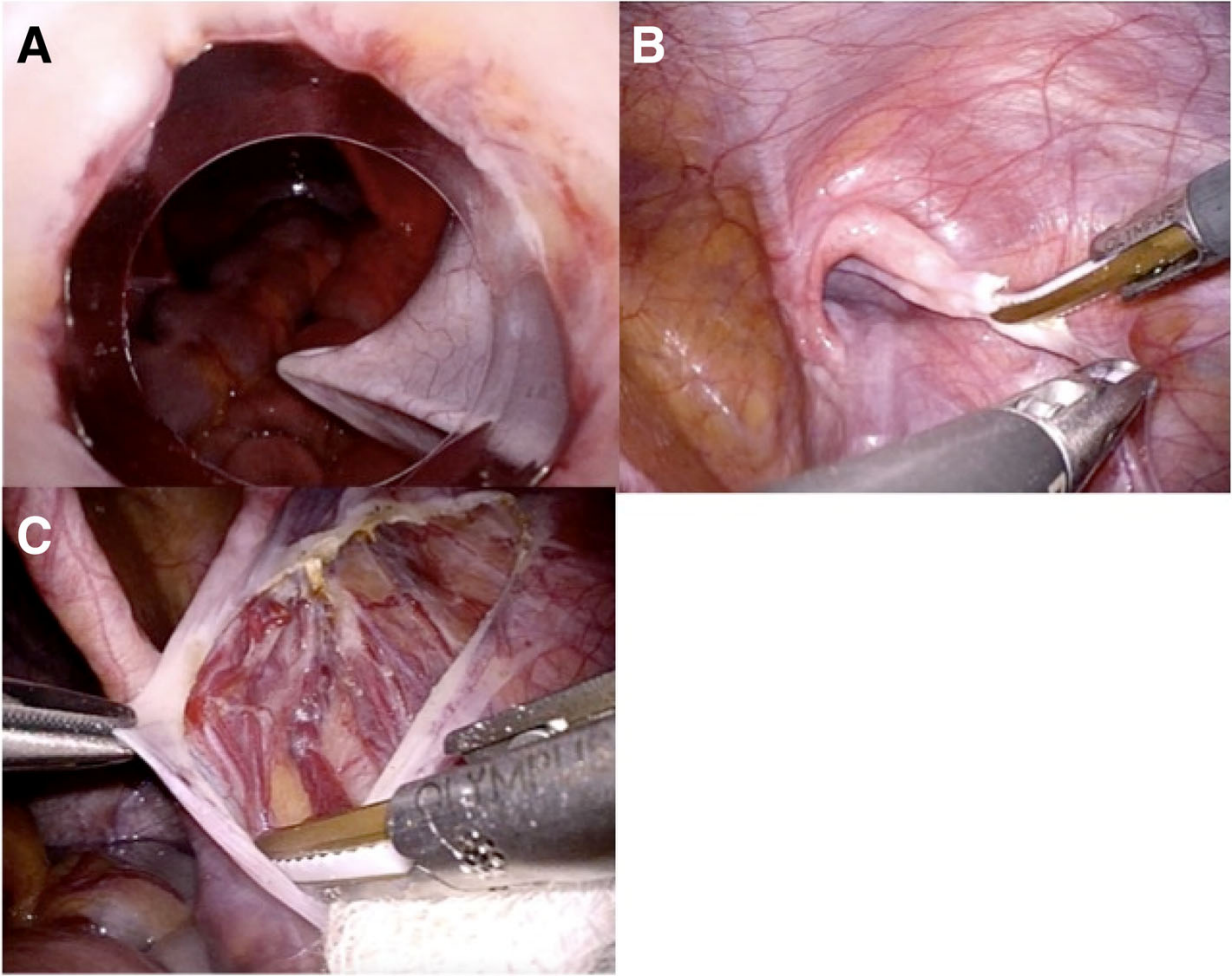
**a** Insertion of trocars was very difficult because of the softness of the abdominal wall. **b** We cut the peritoneum outside of the hernia orifice. **c** A network of veins was present around the spermatic cord and testicular artery

We performed TAPP repair for the right-side inguinal hernia after confirming that no hernia was present on the other side under laparoscopy. After inserting a 12-mm trocar in the umbilicus and identifying the indirect inguinal hernia on the right side and the absence of a hernia on the left side, we inserted 5-mm trocars in the bilateral flank regions. Insertion of the trocars was very difficult because of the softness of the abdominal wall (Fig. 2a), which may have been due to Hunter syndrome. We cut the peritoneum outside of the hernia orifice (Fig. 2b), identified the spermatic cord and testicular artery, and dissected the preperitoneal space with gauze. We found a network of veins around the spermatic cord and testicular artery (Fig. 2c), and special care was required to avoid hemorrhage. We inserted a prosthetic mesh (16.0 × 10.8 cm) into the preperitoneal space and tacked it onto either side of the inferior epigastric artery, transverse abdominal muscle, abdominal rectus muscle, and Cooper’s ligament. The hernia orifice was completely covered, and the peritoneum was closed by suturing. Although the operation involved minimal bleeding, it took 1 h 53 min to complete because the softness of the abdominal wall and the patient’s shorter height made the operative procedure difficult. Although a mild convulsion and subsequent aspiration pneumonia after the operation prolonged his hospital stay, he recovered with appropriate treatments and was discharged on postoperative day 9.

## Discussion

We have herein described an 18-year-old patient with Hunter syndrome treated with the TAPP method for an inguinal hernia. The performance of TAPP repair for inguinal hernias in patients with Hunter syndrome is very rare; this is the first such report according to a PubMed search. Surgeons may avoid TAPP repair for inguinal hernias in patients with Hunter syndrome for various reasons. Most patients with Hunter syndrome develop hernias in early childhood. Lin et al. [3] reported that patients with Hunter syndrome underwent hernia repair in early childhood (mean age, 4.2 years). Surgeons usually select Potts’ method [4], LPEC [5], or laparoscopic direct suture [6, 7] for infants. However, our patient was 18 years old, and few reports have described these methods for inguinal hernias in adults. Additionally, there is no evidence of the usefulness of these methods for young adult patients. Sparkman [8] reported that 15.8% of patients with an inguinal hernia also had a contralateral inguinal hernia. Patients with Hunter syndrome have a high risk of hernia formation, and a wide hernia orifice was present in this case. Therefore, a laparoscopic approach was selected to check the intraperitoneal groin area on the left side.

Because the pathogenesis of inguinal hernia in Hunter syndrome is an indirect hernia and the patient in this case was short for his age, the methods without a prosthetic mesh could be selected. However, the methods without a prosthetic mesh have a higher rate of recurrence than those with a prosthetic mesh for patients who have stopped growing [9–14]. Therefore, we selected TAPP in this case.

Historically, patients with Hunter syndrome had a short life expectancy because of complications such as heart disease and respiratory infections. However, they can now be treated with enzyme replacement therapy with idursulfase. Some trials have proven that enzyme replacement therapy with idursulfase improves the clinical symptoms of patients with Hunter syndrome [15–17] and enables these patients to live longer. Therefore, more young adult patients with Hunter syndrome will require surgery for inguinal hernias in the future. The Japanese Hernia Society recommends operations with a prosthetic mesh for inguinal hernias in young adulthood [18]. Therefore, TAPP repair may be a suitable procedure for young adult patients with Hunter syndrome.

We identified two differences between our patient with Hunter syndrome and normal adults undergoing surgical repair of hernias. One is that the abdominal wall of our patient with Hunter syndrome was lithe and soft, like that of children, making it difficult to insert trocars through the abdominal wall (Fig. 2a). Trocar insertion required lifting of the abdominal wall with strings. The other difference is the presence of a network of well-developed veins around the spermatic cord and testicular artery (Fig. 2c). Because no previous reports have described inguinal hernia repair in patients with Hunter syndrome, whether this vein formation around the spermatic cord and testicular artery is common among these patients remains unclear. If this network of veins is a common feature, gentle manipulation is required when dissecting the preperitoneal space.

Patients with Hunter syndrome may develop many complications. In the present case, a convulsion and pneumonia developed after surgery. However, we predicted these complications based on previously published reports of Hunter syndrome [2–4]. Therefore, we were able to prevent these complications from becoming severe by performing postoperative management in the intensive care unit and ensuring close cooperation with pediatric specialists.

## Conclusion

To our knowledge, this is the first report of the performance of TAPP repair for an inguinal hernia in a young adult patient with Hunter syndrome. TAPP repair might be a useful strategy for hernias in adolescents with Hunter syndrome with appropriate perioperative management and cooperation with specialists.

## Data Availability

Not applicable.

## Abbreviations

LPEC: Laparoscopic percutaneous extraperitoneal closure
TAPP: Transabdominal preperitoneal

## References

[1] Hunter C (1917). A rare disease in two brothers. Proc R Soc Med..

[2] Bach G, Eisenberg F, Cantz M, Neufeld EF (1973). The defect in the Hunter syndrome: deficiency of sulfoiduronate sulfatase. Proc Natl Acad Sci USA..

[3] Lin HY, Chuang CK, Chen MR, Lin SJ, Chiu PC, Niu DM (2018). Clinical characteristics and surgical history of Taiwanese patients with mucopolysaccharidosis type II: data from the Hunter Outcome Survey (HOS). Orphanet J Rare Dis..

[4] Potts WJ, Riker WL, Lewis JE (1950). The treatment of inguinal hernia infants and children. Ann Surg..

[5] Takehara H, Yakabe S, Kameoka K (2006). Laparoscopic percutaneous extraperitoneal closure for inguinal hernia in children: clinical outcome of 972 repairs done in 3 pediatric surgical institutions. J Pediatr Surg..

[6] Bruzoni M, Jaramillo JD, Kastenberg ZJ, Wall JK, Wright R, Dutta S (2015). Long-term follow-up of laparoscopic transcutaneous inguinal herniorrhaphy with high transfixation suture ligature of the hernia sac. J Pediatr Surg..

[7] Darmawan KF, Sinclair T, Dunn JCY (2018). Comparison of laparoscopic and open pediatric inguinal hernia repairs at two institutions. Pediatr Surg Int..

[8] Sparkman RS (1962). Bilateral exploration in inguinal hernia in juvenile patients. Review and appraisal. Surgery..

[9] Butters M, Redecke J, Koninger J (2007). Long-term results of a randomized clinical trial of Shouldice, Lichtenstein and transabdominal preperitoneal hernia repairs. Br. J. Surg..

[10] Liem MS, vanDuyn EB, vanderGraaf Y, vanVroonhoven TJ (2003). Coala Trial Group. Recurrences after conventional anterior and laparoscopic inguinal hernia repair: a randomized comparison. Ann. Surg..

[11] Nordin P, Bartelmess P, Jansson C, Svensson C, Edlund G (2002). Randomized trial of Lichtenstein versus Shouldice hernia repair in general surgical practice. Br. J. Surg..

[12] Tschudi JF, Wagner M, Klaiber C, Brugger JJ, Frei E, Krahenbuhl L, Inderbitzi R, Boinski J, HsuSchmitz SF, Husler J (2001). Randomized controlled trial of laparoscopic transabdominal preperitoneal hernioplasty vs Shouldice repair. Surg. Endosc..

[13] McGillicuddy JE (1998). Prospective randomized comparison of the Shouldice and Lichtenstein hernia repair procedures. Arch. Surg..

[14] Dirksen CD, Beets GL, Go PM, Geisler FE, Baeten CG, Kootstra G (1998). Bassini repair compared with laparoscopic repair for primary inguinal hernia: a randomized controlled trial. Eur. J. Surg..

[15] Muenzer J, Wraith JE, Beck M, Giugliani R, Harmatz P, Eng CM (2006). A phase II/III clinical study of enzyme replacement therapy with idursulfase in mucopolysaccharidosis II (Hunter syndrome). Genet Med..

[16] Muenzer J, Beck M, Eng CM, Giugliani R, Harmatz P, Martin R (2011). Long-term, open-labeled extension study of idursulfase in the treatment of Hunter syndrome. Genet Med..

[17] Muenzer J, Beck M, Giugliani R, Suzuki Y, Tylki-Szymanska A, Valayannopoulos V (2011). Idursulfase treatment of Hunter syndrome in children younger than 6 years: results from the Hunter outcome survey. Genet Med..

[18] Japanese Hernia Society. Japanese hernia society guidelines on the treatment of inguinal hernia. Tokyo, Kaneharashuppan. 2015:70 in Japanese.

